# Barriers and facilitators of communication about *off* periods in Parkinson’s disease: Qualitative analysis of patient, carepartner, and physician Interviews

**DOI:** 10.1371/journal.pone.0215384

**Published:** 2019-04-18

**Authors:** Melissa J. Armstrong, Tara Rastgardani, Anna R. Gagliardi, Connie Marras

**Affiliations:** 1 Department of Neurology, University of Florida College of Medicine, Gainesville, Florida, United States of America; 2 Morton and Gloria Shulman Movement Disorders Centre and the Edmond J Safra Program in Parkinson's Research, Toronto Western Hospital, Toronto, Ontario, Canada; 3 Toronto General Hospital Research Institute, University Health Network, Toronto, Ontario, Canada; Newcastle University, UNITED KINGDOM

## Abstract

**Background:**

Successful patient-physician communication is critical for improving health outcomes, but research regarding optimal communication practices in Parkinson’s disease is limited. The objective of the current study was to investigate barriers and facilitators of communication between persons with Parkinson’s disease, carepartners, and physicians, specifically in the setting of *off* periods, with the goal of identifying ways to improve patient-carepartner-physician communication.

**Method:**

We interviewed persons with Parkinson’s, carepartners, and physicians (specialists and non-specialists) using a semi-structured questionnaire to identify and describe experiences, barriers, and facilitators relating to communication about *off* periods in Parkinson’s disease. We used a qualitative descriptive approach to analyze interview transcripts and compare themes between participating groups.

**Results:**

Twenty persons with Parkinson’s and their carepartners and 20 physicians (10 specialists, 10 non-specialists) participated in interviews. Identified communication barriers included patient-level (e.g. cognitive impairment, reluctance to discuss symptoms), caregiver-level (e.g. caregiver absence), and physician-level (e.g. distraction by technology, lack of appreciation of the burden of *off* periods) factors. Other barriers included the challenging nature of *off* periods themselves. Positive physician characteristics such as empathy, respect, and taking time to listen were major facilitators of communication regarding *off* periods. Persons with Parkinson’s, carepartners, and physicians described using various tools (e.g. home diaries, questionnaires, mobile phone videos) to aid communication regarding *off* periods but participants identified a need for more formal educational materials.

**Conclusions:**

Physicians caring for persons with Parkinson’s can improve communication through more patient-centered practice but there is a need for improved educational tools regarding *off* periods. Further research is needed to identify optimal strategies for communication about *off* periods and preferred approaches for *off* period education.

## Introduction

Numerous studies link patient-physician communication to improved health outcomes [1]. Good communication skills are among the most desired physician qualities described by patients [2]. Showing respect is the aspect of communication most closely tied to physician rating, but patients also want physicians to listen carefully, explain things, provide easy to understand instructions, and spend enough time with them [2]. While over 80% of surveyed adults in the United States (U.S.) indicate that they want their healthcare provider to listen, only 60% say this actually happens and less than half say that their provider asks about their healthcare goals and concerns [3].

Shared decision making (SDM) is a partnership between patients and physicians (and carepartners, where appropriate) which considers patients’ values and preferences alongside the medical evidence to make the best decisions for a given patient in a given scenario. While SDM is lauded as the “pinnacle of patient-centered care” [4], it relies on patient-physician communication which may not be occurring [3]. SDM requires physicians to elicit and understand patients’ values, explain medical evidence including risks and benefits, and partner to integrate those elements to make a decision.

Only 36% of persons with Parkinson’s disease (PwP) and caregivers report satisfaction that their physician “listens to each patient and takes the patient’s concerns seriously” [5]. Multiple studies show that PwP were dissatisfied with the amount of information they received about Parkinson’s disease (PD) and treatment options [5–11]. Most PwP want to make medical decisions on their own or in partnership with their physicians [6, 12], but almost a third of the time, physicians are making decisions for PwP [12, 13] or PwP are less involved in decision making than desired [8]. Even when PwP and caregivers are involved in decision-making, they describe inadequate information for making informed choices [11].

It is also critical to understand patient-physician communication in PD outside the decision-making context, but such research is sparse. PwP emphasize that communication is important to medical care and they desire candor, honesty, understanding and empathy. They value a patient-physician relationship with mutual respect, trust, openness and adequate time [6]. A recent systematic review of communication about *off* periods, however, identified only one study on this topic [14]. In that study, less than half of PwP reported discussing troublesome symptoms at every appointment [15]. The role of carepartners/caregivers in communication in PD is also uncertain. Caregivers commonly support PwP at clinical visits [11, 13] but are engaged by physicians only 40–71% of the time [7, 13].

Given the lack of research regarding communication in PD, we aimed to understand barriers and facilitators of communication between PwP, carepartners, and physicians, specifically in the setting of *off* periods, with the goal of identifying ways to improve patient-carepartner-physician communication. *Off* periods are times when PD symptoms that respond to medication reappear or worsen–usually at the end of a treatment dose–resulting in functional disability. Re-emergent symptoms can include both motor (e.g. stiffness, slowness, tremor) and non-motor (e.g. cognitive, mood) features [16]. *Off* periods were selected as the framing context for the study given the complexity of the phenomenon, its frequency (~40% of patients 4–6 years after starting treatment) [17], poorer associated health-related quality of life [18, 19], and association with treatment decisions [20].

## Materials and methods

### Design

An exploratory research design involving qualitative interviews with PwP, carepartners, and physicians (specialists and non-specialists) was chosen to thoroughly identify and describe communication experiences, barriers, and facilitators [21]. This was part of a larger study investigating *off* period experiences. A qualitative descriptive approach [22] was used to conduct and analyze interviews. This approach aims to explicitly report experiences without intending to use or generate theory. The University Health Network Research Ethics Board provided approval for this study (file number 16–5880). All participants provided written informed consent prior to interview. Consolidated criteria for reporting qualitative research [23] guided the reporting of study findings (S1 Checklist).

### Population and recruitment

#### Patients and carepartners

Convenience sampling was used. U.S.-based PwP and care partners were recruited via emails to the Parkinson Disease Foundation Care Partner 2016 Summit mailing list and posting of study information on Fox Trial Finder (2/13/17-10/16/17), a matching tool connecting PwP to research studies. Interested individuals responded by email, telephone, or through the Fox Trial Finder message system. Inclusion criteria for PwP were a PD diagnosis, presence of *off* periods, U.S. residence, and having a carepartner also willing to be interviewed. Consent forms were emailed to volunteers and returned to the study team prior to the interview. Potential participants had the opportunity to discuss questions by telephone with an investigator. Investigators and participants had no pre-existing relationships.

#### Physicians

Convenience sampling was used to recruit general neurologists and PD specialists working in the U.S. Invitations and consent forms were sent to U.S.-based movement disorder neurologists using the Movement Disorders Society mailing list. Interested participants contacted a study coordinator by telephone or email for further information. General neurologists were invited to participate through the American Academy of Neurology (AAN) members’ online community forum Synapse as well as through a presentation at the general neurology section meeting at the annual AAN conference. Because these strategies were unsuccessful for meeting recruitment targets, most general neurologists were recruited by a recruitment agency (Sermo) using an email introduction sent by the recruitment agency. The agency shared the consent form with interested physicians. Sermo provided signed consent forms to the study team to schedule interviews. General neurologists needed to evaluate at least 10 PwP monthly to meet inclusion criteria.

### Data collection

Investigators performed a preparatory systematic review which showed that (1) *off* periods are among the most troublesome symptoms to PwP, with a broad-ranging impact on patient and carepartner activities, (2) PwP and caregiver understanding of *off* periods is suboptimal, and (3) communication regarding *off* periods is largely unstudied [14]. Based on these results, investigators drafted semi-structured interview guides for PwP, carepartner, and physician interviews covering topics relating to experiences, barriers, and facilitators of communication to address literature gaps (S1 Interview guide). The guides were iteratively revised by authors with movement disorders (MJA, TR, CM) and qualitative research (AG, MJA) experience. Pilot testing occurred with at least one individual representing each of the target groups prior to finalization. Interviews were conducted by phone by a single investigator (TR), a neurologist obtaining movement disorders subspecialty expertise, who was trained and mentored by a PhD with qualitative methodological expertise (ARG). Investigators interviewed PwP and carepartners separately. Target interview length was 30–60 minutes for PwP and carepartners and 20–30 minutes for physicians. Interviews were audio-recorded with participant knowledge and professionally transcribed. Each participant received a $100 prepaid cash card by mail after interview completion except for physicians recruited through Sermo who received $140 based on their standard practices.

### Analysis

Data were organized and analyzed using Microsoft Word and a qualitative descriptive approach [22]. One investigator (TR) independently analyzed transcripts to create a log of codes reflecting emerging themes and sample quotes (open coding). These were reviewed, revised, and discussed with the three other investigators to achieve consensus on emerging themes and expand or merge thematic codes (axial coding). Saturation was determined by discussion and consensus following completion of 20 interviews with each of the three groups. Themes, subthemes, and exemplar quotes were tabulated and summarized. MJA further categorized barriers and facilitators as patient-based, carepartner-based, physician-based, and “other” by referencing categories described in a prior systematic review [24]. Participant checking was not performed.

## Results

Sixty subjects participated (Table 1). All eligible patient and carepartner volunteers were included. Invitations were sent to 999 U.S.-based movement disorders specialists, 20 of whom expressed interest. Thirteen specialists agreed to participate and the first 10 were interviewed. All eligible general neurology volunteers were included. Interviews occurred between March and November 2017. Participating carepartners were mostly spouses (17, 85%; other: son, brother-in-law, close friend). Nine (45%) of participating PwP received care from a general neurologist; the remainder received specialty care. Mean interview duration was 39 minutes for PwP, 33 minutes for carepartners, and 21 minutes for physicians. Themes and select quotes are discussed here; additional quotes are summarized in S1 Appendix.

**Table 1:** Demographic characteristics of interview participants.

	PwP	Carepartners	General neurologists	PD specialists
Total “n”	20	20	10	10
Gender (n, % male)	9 (45%)	9 (45%)	8 (80%)	5 (50%)
Age (years) (mean, SD)	65.1 (8.3)	NA		
Years since diagnosis (mean, SD)	7.8 (4.7)			
Years in practice (mean, SD)			13.8 (7.7)	18.9 (13.5)
Clinic time (minutes) spent with new patients (mean, SD)			52.5 (10.3)	60.5 (5.7)
Clinic time (minutes) spent with follow-up patients (mean, SD)			29.0 (4.9)	29.5 (1.5)

PwP: People with Parkinson’s, PD: Parkinson’s disease, NA: not available, SD: standard deviation

### Barriers to communication

#### Patient-level factors

One of the main barriers described by both PwP and physicians was PwP difficulty describing *off* period experiences:

That general feeling of uneasiness is very difficult to explain to the doctor or anyone for that matter (PwP3).

Not everyone understands what being slow means and I can sort of demonstrate that with my hands and, you know, you can see it. …[but] you don't realize that the fatigue is there, the antsiness is there, the grumpiness sometimes is there … those are the kind of things it's hard to describe (PwP14).

Sometimes people describe this sort of very vague, you know, I just don't feel good in the morning… After lunch I'm this, you know, I need to take a nap and I feel so tired. Or I feel so fatigued, or I feel so weak. So, that's obviously a bit more challenging, cause I mean, who knows? … Sometimes it's very straightforward, they're just saying oh, you know, half an hour before I take my next dose the tremor comes back… I think mostly when people describe their off, or their possible off symptoms as very vague, it makes it much more challenging (MDS4).

General neurologists and specialists described that patient recall of symptoms was an additional barrier (Table 2). Disease-related symptoms such as cognitive impairment, trouble speaking, and anxiety hindered PwP communication regarding off periods:

The biggest challenge is that sometimes just having Parkinson's, my brain don't want to work right and I forget stuff and, you know, I may forget to tell him about, maybe, certain symptoms (PwP13).

PwP admitted that pride, embarrassment, and an unwillingness to admit that something was happening limited what they raised with physicians. While carepartners did not identify many patient-level barriers, they reported that sometimes PwP were unlikely to report symptoms:

I tend to be more upfront and let [the doctor] know what’s going on, where he’s more wait and see, let’s see if it gets worse, when we don’t need to let it get worse, we need to just tell the doctor now (CP5).

The reluctance of PwP to report wearing *off* symptoms was unrecognized by physician participants.

**Table 2:** Barriers to communication about off periods in Parkinson’s disease.

Barrier	Type of Interview Participant			
	PwP	Care- partner	GN	Specialist
Patient-level factors				
Difficulty describing experiences	x		x	x
Cognitive changes	x		x	x
Impaired speech	x			
Anxiety				x
Lack of insight			x	
Lack of English as first language			x	
Pride	x			
Embarrassment	x			
Willingness to admit something is happening	x			
Reluctance to describe symptoms		x		
Symptom recall			x	x
Wanting to give physician a good report	x			
Understanding of PD symptoms			x	
Lack of questioning when physician explanation unclear				x
Carepartner-level factors				
Absence of carepartner at visits	x	x		
Caregiver cognitive impairment			x	
Differing accounts from patient			x	x
Physician-level factors				
Non-appreciation of impact of off periods	x	x		
Lack of interest		x		
Failure to involve carepartner		x		
Distraction by technology	x	x		
Failure to provide education on what to expect	x			
Other factors				
Difficulty linking symptoms to medication timing and distinguishing fluctuating symptoms			x	x
Distinguishing *off* tremor from dyskinesia				x
Difficulty identifying non-motor *off* symptoms				x
Variability of symptoms between individuals				x
Time	x	x	x	
Infrequent visits		x		
Lack of a reliable historian			x	
Lack of a shared vocabulary	x		x	x
Different expectations for medication response				x
Lack of ancillary team support			x	

“x” indicates that the barrier was described by the category of interview participant for that column

PwP: person with Parkinson’s, GN: general neurologist, PD: Parkinson’s disease

#### Carepartner-level factors

PwP and carepartners reported that the lack of carepartners at clinical visits was the main carepartner-related barrier (Table 2). Physicians, on the other hand, reported challenges when carepartners had cognitive impairment themselves or when accounts from PwP and carepartners differed:

You're running up against the difficulty of talking to someone and someone…and the patient saying one thing and the family member saying something else. And, there could be some disagreement and argument about it (GN6).

#### Physician-level factors

Physician-level barriers were described by PwP and carepartners but not by physicians themselves (Table 2). PwP described frustration that physicians do not appreciate the degree to which *off* periods impact daily life.

I guess maybe just drawing the line from the fact that I have these off periods to the fact that I think they're a major impediment to my life, and I think he thinks of them as just normal for Parkinson's patients… It's hard for me to feel heard because what I really would like to do is go back to work, and I can't go back to work when I'm having off periods. And I think he's not thinking about me going back to work… (PwP2).

Carepartners felt that physicians do not investigate the depth of patient *off* period experiences:

They're certainly not asking the level of questions you are, that like, you know, what is the impact and extent and severity of these off periods (CP2).

Carepartners reported lack of physician engagement of carepartners in clinic visits as a barrier:

Well, I think the neurologist should inquire a whole lot more of me. I mean, they obviously need to direct themselves towards my husband as a person with Parkinson’s and all of that kind of thing, but I think they miss and have missed a whole bunch of data and information, and it took me a couple of years, not because I’m not assertive or anything, but it was kind of a new experience to be my husband’s spokesperson. But I used to sit there and think they aren’t getting half the story just listening to how my husband perceives what’s happening with him, and I thought it was odd that, I thought it was really odd that the reaction to the person who is with him 24/7 isn’t more important to these neurologists (CP1).

Both PwP and carepartners described challenges from physicians distracted by technology:

I feel that he's very distracted by the technology that they use, those computers. And I feel like he has, like, his checklist, and he really does need to get through that before he can really focus on me… They're computerizing everything and he is not… he's not a computer guy… At best he's a two-fingered typist and he has to pay complete attention to the computer or me. He can't do both (PwP2).

#### Other factors

The complicated nature of *off* periods is itself a barrier to communication. Physicians reported difficulty establishing whether symptoms related to PD, medication timing, or something else.

You know, patients just say they don't feel well all day long. And, so, this is hard to kind of say, "Well, was there a time when it was really different? And, how does it relate to my doses? Is it the beginning of my dose? The end of my dose? And kind of arbitrary?" (GN7).

Movement disorder specialists described the difficulty of distinguishing tremors during *off* periods from peak dose dyskinesias:

A lot times patients misinterpret their dyskinesia as tremor and that can be confusing because when they’re really on they think they’re off and they’re worried about that movement when it’s really an on phenomenon (MDS10).

Specialists also reported challenges in identifying the presence of non-motor *off* symptoms:

…So much of the time the off-periods are–so there are psychological symptoms, and if they're associated with obvious severe motor symptoms, then that’s not hard to ferret out. But so many patients don’t understand that the motor wearing off might be mild and the emotional symptoms might be much more severe (MDS7).

Related to these challenges is the lack of shared vocabulary about *off* periods, described by both PwP and physicians.

I also learned what an off period is, and that's what I'd been describing to my doctor as just the unevenness, I called it the unevenness before (PwP2).

Helping PwP understand what to expect, though, can be challenging:

All symptoms vary quite substantially between individuals, so it's not sort of where I can say, here's a list of things, you know, more tremor or feeling slower or stiffer or things like that, because I think that people experience off differently (MDS2).

Clinical visit time was a barrier described by PwP, carepartners, and general neurologists, but not specialists. Lack of ancillary support (e.g. advance practice providers [APPs]) was a barrier for small general neurology practices.

### Facilitators of communication

Identified patient- and carepartner-level facilitators were rare, though all groups agreed that having a carepartner present at visits was helpful (Table 3).

**Table 3:** Facilitators of communication about off periods in Parkinson’s disease.

Facilitator	Type of Participant			
	PwP	Care- partner	GN	Specialist
Patient-level factors				
Openness with physician about symptoms, experiences		x		
Carepartner-level factors				
Presence of carepartner to clarify symptoms, serve as a second person to hear physician	x	x	x	x
Physician-level factors				
Empathy, respect	x			
Taking time to listen	x	x	x	
Interested, curious	x	x		
Asking questions about impact on daily life		x		
Body language (e.g. eye contact)		x		
Appreciation of individual experiences, addressing individual concerns	x	x		
Separating conversation from data entry		x	x	
Education, counseling		x	x	x
Other clinical factors				
Continuity of care			x	
Face-to-face conversations	x	x		
Asking consistent questions at each visit			x	
Using teach-back method			x	
Allied health/advance practice providers			x	x
Patient portal for communication between visits	x	x		
Tools to help symptom reporting				
Creating pre-visit agenda	x	x		
Questionnaires			x	
Diaries	x		x	x
Developing a shared vocabulary				x
Home videos of symptoms	x		x	
Wearable technology				x
Levodopa challenge				x
Educational tools				
Hand-drawn graphics			x	x
Informal online videos			x	
Online forums			x	
Local PD classes			x	
Education programs led by advance practice/ allied health professionals				x
Video education (described as desirable)			x	x
Handouts (described as desirable)			x	x

“x” indicates that the facilitator was described by the category of interview participant for that column

PwP: person with Parkinson’s, GN: general neurologist, PD: Parkinson’s disease

#### Physician-level factors

PwP valued physician traits such as empathy and respect.

Parkinson’s is a very individual disease. And so you need somebody that’s going to, you know, care about that and respect that and listen to you when… there’s something really concerning you. I wouldn’t bring this up if it wasn’t important (PwP8).

Physician behaviors such as listening to PwP and carepartners, demonstrating interest and curiosity, and appreciating individual experiences were also identified facilitators:

Well, the fact that the doctor actually sat down and took the time to listen and hear what I had to say, really listen to all of the symptoms I was conveying, not just looking at the tremors or trying to figure out the small handwriting or whatever. He was listening to what I was feeling relative to smell and taste and hearing and unsteadiness. He was listening to all of it (PwP3).

She actually sits and listens, and she doesn’t try to make you think that something else is happening (CP5).

You know, the doctors definitely are open to talking to both of us and, you know, I certainly get the feeling that he’s as interested in what I observe as what my husband observes (CP12).

General neurologists described the importance of taking time to listen to PwP and carepartners:

I think trying to be cognizant of not being rushed when I'm actually in there… When I see a patient I actually don’t–even though I have an electronic medical record–I actually don’t type or work on EMR when I'm seeing a patient. So, trying to be present with them and having a conversation with them versus trying to do my data entry at the same time I'm talking to them (GN2).

#### Other clinical factors

General neurologists noted the value of longitudinal relationships over time, using the same questions each visit to facilitate patient understanding, and recognizing the importance of individual preferences (Table 3). PwP and carepartners described the value of in-person visits, though they also appreciated patient portals for communication between visits.

I still think the face to face conversations where I can ask him questions and he can give me answers or he can, you know, actually grab my arm and feel for rigidity or see how I walk across the room, I think those are the kinds of things that I benefit from the most (PwP3).

#### Tools to help symptom reporting

Interviewees described various tools as facilitators of communication regarding *off* periods. PwP and carepartners reported creating a list of concerns prior to clinic visits to guide discussion, sometimes using questionnaires to guide that preparation (Table 3). PwP also reported that using diaries and schedules was helpful if things weren’t going well. One PwP described using a video to help his physician understand what was happening, but one carepartner felt that his loved one might feel “invaded” if he videotaped *off* periods.

General neurologists and specialists described using home videos and patient diaries to clarify patient symptoms, though several specialists described abandoning diary use given difficulty explaining instructions and overwhelming amounts of data. General neurologists also reported using questionnaires to streamline clinic interviews. Specialists described using levodopa challenges and wearable technology. Several specialists described the importance of intentionally developing a shared vocabulary:

I sit down with the patient and I have just a blank sheet of paper and big lettering, and I put what’s on in one column and what’s off in another column… I’ll say, when your medicine is not working, what [are] you like? You know, if they do have tremor, and… right off the bat they’ll say something, and so I write that down. And they might say… I'm sitting there at my desk and I'm getting a lot done, but then I stand up and my feet won’t go, and that’s why I know, oh darn it, I missed my dose two hours ago… So then I write down, okay, your feet won't move, then I use their terminology and I write that down in the off column. And so then they take that piece of paper home and when they're filling out their diary, then they can look and say, now which one do I fit–the on or the off column–right now? So that’s another tool (MDS7).

I give them kind of language because I think the most important thing is a shared language (MDS8).

#### Educational tools

Developing shared vocabulary was viewed as both a communication strategy and an educational tool. Other educational strategies included taking time to educate patients about *off* periods, often by sketching graphics (Table 3).

I'll draw a little, a little diagram for them, you know, where it's like being in the right window versus having too much and then having dyskinesias and then too little and feeling off (MDS5).

One specialist noted the particular importance of educating PwP about non-motor fluctuations. A general neurologist described using the teach-back method to ensure understanding. General neurologists reported using informal online videos to demonstrate symptoms and referring patients to online forums or local support groups. Numerous movement disorders specialists described the value of having allied health professionals and APPs to provide education informally or via scheduled training sessions.

Both general and specialist neurologists voiced interest in having video resources to teach people about *off* periods. Movement disorders specialists felt that having an educational page to send home with PwP would be helpful, whereas one general neurologist thought that a pamphlet would not be much use given the lack of associated visuals.

## Discussion

PwP, carepartners, general neurologists, and movement disorder specialists identified numerous patient-level, carepartner-level, and physician-level barriers and facilitators to communication regarding *off* periods. In a prior study of advanced PD, PwP, carepartners, and physicians also identified lack of information, poor neurologist support, patient cognitive impairment, lack of caregiver involvement, and PwP-caregiver disagreements as barriers to decision-making [11], consistent with current results. The barriers identified in this study are also consistent with those reported by patients across diseases: patient uniqueness/variability, cognitive/physical impairments, poor physician listening, insufficient information/education, use of medical terminology, lack of time, lack of decision support, and differing expectations between patients and physicians [24]. While many of these barriers are relevant outside PD, the challenging nature of *off* periods is a unique barrier affecting many aspects of PwP-carepartner-physician communication. PwP described difficulty putting their *off* symptoms into words. Physicians described challenges correlating symptoms with medication dosing and identifying if there is a treatable fluctuation. Both parties highlighted the lack of a shared vocabulary.

Facilitators of communication described in this study and in the literature include presence of a caregiver for decision support, continuity of care, adequate time, respect, physicians taking time to listen, good communication, provision of information about options, and written decision support [11, 24]. Strategies to supplement physician teaching and in-person visits (e.g. use of APPs, patient portals) were also facilitators of communication regarding *off* periods (Table 3). Successful communication regarding *off* periods, though, necessarily includes facilitators specific to this phenomenon. Pre-visit preparation and questionnaires were identified as facilitators of communication in our study, consistent with prior findings that using wearing off questionnaires was helpful in identifying fluctuations (particularly motor) [25, 26]. Diaries, home videos, levodopa challenges, and wearable technology have utility in select circumstances, but patient/carepartner comfort with selected approaches and physician resources for conducting and/or interpreting these assessments, including the time needed to evaluate large amounts of data, may limit these approaches. Additionally, while 87% of PwP described interest in recording information to monitor symptoms in a survey, only 49% were actually doing so [27].

Our results highlight that lack of patient/carepartner education regarding *off* periods, lack of shared understanding of the concept between PwP and physicians, and limited physician appreciation of the personal impact of *off* periods are important modifiable communication barriers. These results are consistent with prior PD research demonstrating lack of education and understanding as barriers to PD care [5–10]. Physicians described the value of educational programs, classes, and online forums, but used verbal education and hand-drawn graphics in clinical encounters. Physicians desired video educational tools and handouts regarding *off* periods to improve education and communication, consistent with a known need for better patient education in PD [28]. These findings suggest an opportunity for Parkinson societies, patient organizations, centers, and physicians to develop *off* period video and print educational tools for use within clinical encounters, in community settings, and for patient and carepartner self-education. Subsequent to the conduct of this study, the Parkinson’s Foundation published “Managing PD Mid-Stride,” a booklet with text and figure-based education regarding *off* periods that addresses some of the needs identified by study participants [29]. Educational tools can potentially improve health related quality of life in PD [30] and medication adherence [31].

While it is not surprising that physicians neglecting to probe the daily impact of *off* periods, failure to engage carepartners in clinical visits, and distraction by technology impeded communication, physicians in our study appeared largely unaware of the ways they impeded communication about *off* periods. Opportunities for physicians to improve communication include allowing more time, involving carepartners, active listening, showing interest, asking about the personal impact of wearing *off*, avoiding distractions from technology, and providing education about *off* periods and what to expect (Table 4). These are common-sense approaches for patient-centered medical care, but they are important to specifically identify for clinicians given the experiences reported by participants in this study and studies showing PwP dissatisfaction with communication and the information they receive [5–11]. Identifying these facilitators also has implications for hospital system and clinic planning: clinicians need adequate time to assess patients and query the daily impact of *off* periods, clinical rooms designed to facilitate interactions of patients, families, and clinicians and seamlessly integrate technology, ancillary staff to support assessments and education, and opportunities for education (Table 4). Patients and carepartners can also take steps to improve communication with their medical team (Table 4).

**Table 4:** Practical approaches for improving communication regarding *Off* periods.

Population Associated with Suggested Strategies	Approaches
Patients, carepartners	- Keeping track of symptoms between visits to enhance recall - Having carepartners, others at clinical visits - Pre-visit preparation (e.g. creating an agenda, discussing topics to raise, completion of pre-visit questionnaires) - Describing symptoms and challenges with honesty - Targeted use of strategies such as diaries, home videos - Self-education through asking questions of medical team, attending PD classes, published resources (print, online, video) - Asking questions when something is unclear
Clinicians	- Demonstrating empathy, respect, interest, curiosity - Taking time to listen to patients, carepartners at visits - Asking about *off* periods at every visit - Probing the impact of *off* periods on daily function - Acknowledging the uniqueness of each individual’s concerns - Involving carepartner at clinical visits - Separating conversation from data entry - Intentionally creating a shared *off* period vocabulary with patients, carepartners - Providing verbal and print/online education regarding *off* periods and what to expect - Allowing time for patients and carepartners to both respond to questions and ask questions - Use of teach-back methods to ensure understanding - Targeted use of strategies such as diaries, home videos
Hospitals, Clinics	- Ensuring continuity of care across longitudinal clinical visits - Allotting sufficient time for clinical encounters - Creating clinical spaces enabling communication without interference from technology - Hiring additional staff (e.g. allied health, advance practice providers) to provide additional assessments and education - Providing mechanisms for communication between visits (e.g. patient portals) - Providing educational programs (e.g. classes, symposia) and print educational materials regarding *off* periods

### Limitations

This study likely enrolled highly engaged PwP and carepartners and thus may not represent barriers and facilitators for patients who are less active in seeking information about their PD. Recruitment through an online research matching tool will underrepresent the views of those who do not use the internet as a communication method, though it is notable that even these connected PwP describe numerous communication barriers. Patients had to recognize that they have *off* periods to meet inclusion criteria, so the study lacks the views of PwP who have *off* periods but don’t recognize them. General neurologists had to see at least 10 PwP monthly to participate, so this study did not capture the views of general neurologists with limited PD experience. All participating subjects were U.S.-based and this limits generalizability to other medical contexts. Response to physician recruitment strategies was low, consistent with other survey- and interview-based studies but also affecting generalizability.

### Conclusions

Identified barriers and facilitators to communication between PwP, carepartners, and physicians regarding *off* periods include components related to communication generally and challenges relating to *off* periods in particular. Physicians caring for PwP can improve communication through more patient-centered practice but there is also a need for improved educational tools regarding *off* periods. Successful tool development will require PwP and carepartner involvement. Opportunities also exist for PwP, carepartners, and clinics/hospital systems to contribute to improved communication. Further research is needed to identify optimal strategies for communication about *off* periods and preferred approaches for *off* period education.

## Supporting information

S1 Checklist COREQ checklist. COREQ 32-item checklist outlining the page where each element of qualitative research is reported.
(DOCX)

S1 Interview guide Semi-structured interview guides for PwP, carepartners, and physicians. This file includes the three semi-structured interview guides used for the study.
(DOCX)

S1 Appendix Qualitative coding. Coding tables for barriers and facilitators to communication about off periods for PwP, carepartners, and physicians.
(DOCX)
