# Supplementary material for: Barriers and facilitators of communication about off periods in Parkinson’s disease: Qualitative analysis of patient, carepartner, and physician Interviews
Source: PLoS One. 2019 Apr 18;14(4):e0215384. doi: 10.1371/journal.pone.0215384 (PMC6472878; doi:10.1371/journal.pone.0215384)
Supplement: S1 Interview guide — This file includes the three semi-structured interview guides used for the study. (DOCX) [file pone.0215384.s002.docx]

**S1_Interview Guide. Semi-structured interview guides for (1) persons with Parkinson’s (PWP), (2) carepartners and (3) physicians (general neurologists and specialists)**

**1. Interview guide for Persons with Parkinson’s [version date May 2 2017]**

**Opening Remarks**

Hello <name>. Thank you for agreeing to speak with me. My name is <blank and I work as a blank at blank>. Today we shall be talking about OFF periods. OFF periods are temporary episodes of poor mobility and other symptoms in Parkinson’s disease, when medications are not working effectively. These symptoms include fluctuating fatigue, slow or stiff movement, problems with bodily functions, inability to concentrate, and anxiety, to name a few. OFF periods are common in Parkinson’s disease, and can affect quality of life.

In this study, we would like to learn more about how you view OFF periods, how they impact your life, and how you discuss it with your doctor. Before we begin, I’d like to confirm that you experience OFF periods as part of your diagnosis of Parkinson’s disease.

(If yes) – Do you have any questions before we begin?

(If no) – Not eligible for interview, terminate

**Basic demographic data**
Firstly, I’d like to get some basic information about you and your diagnosis of Parkinson’s disease.

How old are you?

When were you diagnosed with Parkinson’s disease?

What kind of doctor treats you for Parkinson’s disease? (Prompts: A family physician, internist, general neurologist, or movement disorders specialist?)

**Experience**

Please describe your OFF symptoms and episodes.
Additional prompts:

- How do you know when you are having an ‘OFF’ period?
- Which symptoms are most common?
- When and how frequently do they occur?
- If only motor symptoms described, ask about non-motor/affective component (and vice versa)

**Impact**

How do OFF periods impact you?

Additional prompts:

- Well-being, ability to perform usual activities of daily living, self-confidence, relationships, social activity, employment
- What aspects of your OFF time impact you the most?
- If further prompt is needed: for example, some patients are most impacted by unpredictable OFF time, some patients complain of greater difficulty walking, while others feel most disabled by anxiety - what parts of your OFF time impact you the most?

**Communication**

How do you think communication with your doctor regarding OFF symptoms impacts your medical care?

How do you discuss OFF with physicians?

Additional prompts:

- When in the course of the disease did you begin to talk about OFF with your doctor?
- Who broached the subject of OFF? (e.g., you, care partner, doctor)
- Does your doctor ask about OFF symptoms at each visit?
- Have all your questions or concerns about OFF been addressed by physicians?
- What types of physicians, with what type do they communicate the most?
- In what setting(s) do you communicate with your physician(s)?

**Barriers & Challenges**

What is the biggest barrier or challenge of communicating about OFF with your doctors?

Why do you think this/these barriers exist?

Additional prompts:

- Physician knowledge, attitude, time, type of physician
- Personal/individual need and preferences
- When you see your doctor do you always recall what you want to discuss?
- Do you have enough time to discuss your questions (about OFF) at each visit?
- Do you feel that your questions are answered?

**Facilitators & Interventions**

What (information, guidance, tools or strategies) would help you to communicate about OFF with doctors?

Additional prompts:

- Education for doctors? Education for patients or carers?
- Pre-consultation question prompt lists?
- Do you use any tools to help monitor your OFF time, such as a written or electronic diary?
- How does using this tool impact your communication with your doctor?

**Knowledge**

Where have you acquired information about OFF?

How did that information help you?

Additional prompts:

- Sources:
  - library, web site, foundation, support group, family, friends, physicians
- Help:
  - Understand OFF?
  - Help you to self-manage OFF symptoms/episodes?
  - Help you cope with OFF and minimize impact on well-being/daily function
- What was most useful source/form of information?

**Conclusion**

Those are all my questions for today.  Do you have any other comments about ‘OFF’ periods that you would like to share?

Thank you again for taking the time to speak with me.

**2. Interview guide for Carepartners of Persons with Parkinson’s [version date May 2 2017]**

**Opening Remarks**

Hello <care partner name>. Thank you for agreeing to speak with me. My name is <blank and I work as a blank at blank>. Today we shall be talking about OFF periods. OFF periods are temporary episodes of poor mobility and other symptoms in Parkinson’s disease, when medications are not working effectively. These symptoms include fluctuating fatigue, slow or stiff movement, problems with bodily functions, inability to concentrate, and anxiety, to name a few. OFF periods are common in Parkinson’s disease, and can affect quality of life.

In this study, we would like to learn more about how you and your loved one view OFF periods, how they impact your life, and how you discuss it with your loved one’s doctor. Before we begin, I’d like to confirm that your loved one who has Parkinson’s disease does experience OFF periods.

(If yes) – Do you have any questions before we begin?

(If no) – Not eligible for interview, terminate

**Experience**

In one to two sentences, please describe to me your understanding of “OFF” periods in Parkinson’s disease.

Please describe your experience of when your loved one has “OFF” symptoms or episodes.

Additional prompts:

- Which symptoms are most common?
- If only motor symptoms described, ask about non-motor (and vice versa)
- When and how frequently do they occur?

**Impact**

How do your loved one’s ‘OFF’ periods impact you?

Additional prompts:

- Well-being, ability to perform usual activities of daily living, self-confidence, relationships, social activity, employment

**Communication**

How do you think communication with your doctor regarding OFF symptoms impacts your loved one’s medical care?How are you engaged in OFF discussions with your loved one’s doctors?

Additional prompts:

- When was the topic of OFF broached? Who brought it up? (e.g., you, physician, your loved one)
- Are OFF symptoms discussed at each visit?
- Have all your questions about OFF been addressed by physicians?
- In what way do you wish your communication with your loved one’s physicians was different?
- In what settings are you communicating with doctors (e.g., at clinic visits, over the phone if your loved one is not feeling well)?
- What types of physicians, with what type of physician do you communicate the most?

**Barriers & Challenges**

What is the biggest barrier or challenge of communicating about OFF with your loved one’s doctors?

Why do you think these barriers exist?

Additional prompts:

- Physician knowledge, attitude, time, type of physician
- Personal/individual need and preferences
- Are you directly addressed during clinic visits? Is your input obtained by the doctor?
- When you see your doctor do you always recall what you want to discuss?
- Do you have enough time to discuss your questions (about OFF) at each visit?
- Do you feel that your questions are answered?

**Facilitators & Interventions**

What information, guidance, tools or strategies would help you to be engaged or to communicate about OFF with doctors?

Additional prompts:

- Education for doctors? Education for patients or care partners?
- Pre-consultation question prompt lists?
- Do you, or your loved one, use any tools to help monitor your OFF time, such as a written or electronic diary?
- How does using this tool impact your communication with your loved one’s doctor?

**Knowledge**

Where have you acquired information about OFF?

How did that information help you?

Additional prompts:

- Sources:
  - library, web site, foundation, support group, family, friends, physicians
- Help:
  - Understand OFF?
  - Help you to manage your loved one’s OFF symptoms/episodes?
  - Help you yourself cope with OFF?
- What was most useful source/form of information?

**Conclusion**

Those are all my questions for today.  Do you have any other comments about ‘OFF’ periods that you would like to share?

Thank you again for taking the time to speak with me.

**3. Interview guide for Physicians Caring for Persons with Parkinson’s [version date May 13 2017]**

**Opening Remarks**

Hello <physician name>. Thank you for agreeing to speak with me. My name is <blank and I work as a blank at blank>. As you may know, “OFF” periods are temporary periods of poor mobility and other non-motor symptoms that are common in Parkinson’s disease and are associated with poorer quality of life.

In this study, we aim to better understand communication about OFF periods between patients, care partners, and physicians. Before we begin, do you have any questions?

**Basic demographic data**

Firstly, I have a few basic questions about your practice:

Are you a general neurologist or movement disorders specialist?

How much time do you typically have to assess a patient with Parkinson’s disease as:

1. A new patient?
2. A follow up patient?

**Physician perspective of impact**

In your clinical experience, what are the major ways or patterns in which ‘OFF’ symptoms impact patients and their care partners?

**Knowledge (implied in explanation of OFF to patients)**

How would you explain the concept of ‘OFF’ symptoms to patients?

**Communication**

At what point in the disease do you broach this topic (for example, at diagnosis, at first follow up visit, etc.)?

What do you ask of patients when assessing ‘OFF’ symptoms?

Prompts:

- do you have a standard process and/or information materials?
- do you tailor this to patient characteristics?

**Barriers & Challenges**

What factors challenge communication about OFF with patients and/or care partners?

Why do you think this/these barriers exist?

Prompts:

- clinical time constraints
- lack of information resources/guidance (guidelines, decision aids)
- lack of an interdisciplinary team including nurses/other allied staff
- patient understanding
- conflicting reports between patients and carepartners – if so, how whose report do you follow/how do you resolve the conflicting information?
- refreshing your own knowledge or skills

**Facilitators and interventions**

What would help you to communicate about OFF with patients and/or care partners?

Additional prompts:

- Education for doctors? Education for patients or carers?
- Pre-consultation question prompt lists?
- Patient coaches?
- nurses/allied health care staff?

**Conclusion**

Those are all my questions for today.  Do you have any other comments about ‘OFF’ periods that you would like to share?

Thank you again for taking the time to speak with me.
