# Supplementary material for: Barriers and facilitators of communication about off periods in Parkinson’s disease: Qualitative analysis of patient, carepartner, and physician Interviews
Source: PLoS One. 2019 Apr 18;14(4):e0215384. doi: 10.1371/journal.pone.0215384 (PMC6472878; doi:10.1371/journal.pone.0215384)
Supplement: S1 Appendix — Coding tables for barriers and facilitators to communication about off periods for PwP, carepartners, and physicians. (DOCX) [file pone.0215384.s003.docx]

**S1_Appendix**: Qualitative Coding With Regards to Barriers and Facilitators to Communication about Off Periods

| **Table 1. PWP-reported barriers to communication regarding OFF periods** | | |
| --- | --- | --- |
| **Theme** | **Sub-theme** | **Exemplar quotations** |
| Difficulty describing OFF symptoms | Nature of affective non-motor off symptoms make them difficult to articulate | - That general feeling of uneasiness is very difficult to explain to the doctor or anyone for that matter. PwP3 - I think in part sort of the antsiness, the unsettlement. Being slow, I think, not everyone understands what being slow means and I can sort of demonstrate that with my hands and, you know, you can see it. …[but] you don't realise that the fatigue is there, the antsiness is there, the grumpiness sometimes is there … those are the kind of things it's hard to describe. PwP14 |
|  | Off symptoms are difficult to articulate because they are novel to the PwP | - It was a little challenging on how they felt like; I wasn’t too sure how to explain it, because it was new to me. PwP11 |
| Others cannot understand | Off symptoms are difficult to articulate because non-PwP cannot relate directly to the same quality of discomfort | - I think for me sometimes it's hard to describe what the symptom is and hard to make sure that I'm conveying, you know, the degree of…discomfort . It's not really pain… it's not something like oh I smashed my finger on the door and everybody sort of knows what that feels like to smash your finger in the door. But I think sometimes if you don't have Parkinson's it's hard to explain to somebody that doesn’t have Parkinson's… because being slow, I think, not everyone understands what being slow means. PwP14 - I can try to tell somebody [about slowness], but I don’t think they can understand. If I talk to other people with Parkinson’s they seem to understand pretty quickly what I’m talking about. PwP20 |
| Lack of shared vocabulary | Off symptoms are difficult to convey because the **language** used by PwP can be different to clinical terms | …I also learned what an off period is, and that's what I'd been describing to my doctor as just the unevenness, I called it the unevenness before. PwP2 |
| PD symptoms themselves are a barrier to PwP’s ability to communicate ‘off’ symptoms | Slowness of speech is a barrier to communication for PwP | - One of the barriers is actually the disease itself, because it affects how quickly you can talk. PwP20 |
|  | Cognitive changes are a barrier to communicating ‘off’ symptoms for PwP | - The biggest challenge is that sometimes just having Parkinson's, my brain don't want to work right and I forget stuff and, you know, I may forget to tell him about, maybe, certain symptoms PwP13 - The disease itself [is a barrier], because it affects how quickly I can think [during appointments]. PwP20 |
| PwP experiences | Embarrassment | - Embarrassment. Sometimes you don’t like to say what’s going wrong with you. Well, you start by saying, “I have this – I'm having this issue.” And then like I say, sometimes the more you know, the worse it is and you don’t want to like talk about something too much. It’s depressing. PwP8 |
|  | Admission that something is happening | - [The biggest barrier in communication is to]…admit to them and then to myself really, because it’s like I can’t believe this is really happening, but it is really happening. PwP11 |
|  | Pride | - It’s probably the man in me, because I don’t want to be looked at as a … I don’t want to be so dependent. Stand on your own two feet and bull your way through it. PwP4 - I guess I’m just too proud to bring anything up, like I’m not really sick, you don’t want to say that you’re sick and you don’t want to bring anything up that’s wrong with you, so it’s … Because my wife told the M.D. that I had something wrong with me and he referred me to the neurologist because…I just don’t bring things up. PwP6 |
|  | Desire to give a good report | - Maybe when I go see him I think I must admit that I want to give him a good report. PwP16 |
| PwP may not have a care partner available at appointments [implied] | PwP may appreciate second set of ears at appointments but care partner is unable to attend | - I've asked him [my care partner] to go with me or if he wants to go with me but he has a big problem with doctors and hospitals and so it bothers him to go in with me. PwP13 |
| PwP can feel that bringing up symptoms is an imposition on the doctor |  | - … To not waste his time, it's funny I guess because I've been in that situation, I don’t know … You know I was a paediatrician, but I am very sensitive to the time constraints and the fact that I should be able to figure this stuff out myself. And you know that his time is better used with patients that maybe need more guidance. PwP6 |
| Impact of off periods is poorly understood by and difficult to convey to others, including physicians | Extent of impact on lifestyle can be unacknowledged/unaddressed by physicians | - I guess maybe just drawing the line from the fact that I have these off periods to the fact that I think they're a major impediment to my life, and I think he thinks of them as just normal for Parkinson's patients. And so. . . it's hard for me to. . . it's hard for me to feel heard because what I really would like to do is go back to work, and I can't go back to work when I'm having off periods. And I think he's not thinking about me going back to work…He's not, he doesn't draw the thread to me really wanting to go back to work. PwP2 |
|  | Extent of impact of cognitive aspects of off periods on PwP sense of self is poorly understood/difficult to convey | - I don’t know that they really can understand how much it affects my mental status and my intelligence, you know, I feel like it’s just kind of slipping away PwP18 |
| Uncertainty regarding what to expect |  | - If someone doesn't say to you, look, you're always going to have off periods, then I'm always going to be banging my head against this thing about where I'm trying to get them shut off. And I -- if someone would just tell me no, you're always going to have them, don't worry about it, then I'd just go with it. PwP2 |
| Lack of time with physician | Some PwP do not feel they have adequate time with the physician to communicate adequately | - She knew me, but yet she was always fast, she talked to you, but she talked to you fast and then she was gone...20 minutes at the most and then she was gone. PwP11 |
|  | It is difficult to convey information about a several month period in a single clinic visit | - And we were just talking about that yesterday, about how within like 30 minutes she tries to find out what’s been happening for the past four months. And that is hard to communicate. PwP8 |
| Technology can be a barrier to communication in clinical visits | Technology that directs the physicians’ attention away from listening is identified as a barrier by PwP | - Yeah, I really do. I feel that he's very distracted by the technology that they use, those computers. And I feel like he has, like, his checklist, and he really does need to get through that before he can really focus on me. That's true, I do feel that way. They're computerising everything and he is not. . . he's not a computer guy. He's not a -- at best he's a two-fingered typist and he has to pay complete attention to the computer or me. He can't do both. PwP2 |

| **Table 2. PWP-reported facilitators to communication regarding OFF periods** | |
| --- | --- |
| **Theme** | **Exemplar quotations** |
| Physician respect | - I do think Parkinson’s is a very individual disease. And so you need somebody that’s going to, you know, care about that and respect that and listen to you when you have your – or if there’s something really concerning you. I wouldn’t bring this up if it wasn’t important. So them realizing that and responding to that. PwP8 |
| Taking time to listen | - Well, the fact that the doctor actually sat down and took the time to listen and hear what I had to say, really listen to all of the symptoms I was conveying, not just looking at the tremors or trying to figure out the small handwriting or whatever. He was listening to what I was feeling relative to smell and taste and hearing and unsteadiness. He was listening to all of it. PwP3 - Well, she listens to what I’m talking about and she addresses it; she doesn’t try to skirt around it or, you know, just not interested. PwP5 - He listens. Listens and makes suggestions and listens to your feedback, he's just a very good clinician...The listening, he comes in and he sits down and he'll ask how things are going and then he'll ask specific questions you know … We did this last time, I mean how did this work out you know, that kind of thing. PwP6 - I do think Parkinson’s is a very individual disease. And so you need somebody that’s going to, you know, care about that and respect that and listen to you when you have your – or if there’s something really concerning you. I wouldn’t bring this up if it wasn’t important. So them realizing that and responding to that. PwP8 |
| Physician curious to hear about PWP experiences | - I feel like I can tell him just about anything and I think I have. He's very curious about how I'm doing PwP17 |
| Empathy | - There was some empathy that recognized the patient was in a different head space and it was a head space that he should address, which he did. PwP 19 |
| Face-to-face conversations | - And we have a conversation; I just tell her what they look like and how they are and have a conversation about whether or not at this point we need to do anything. PwP17 - It's a one-on-one -- well first they do a basic medical, you know, blood pressure, all that kind of stuff, and then I go in and sit down with him and we -- it's mostly conversational…I think it's pretty much a matter of sitting down, he's like, "Okay, so what's going on?" and I tell him what's going on, PwP17 - It [pre-appointment questionnaire] may help a little. I still think the face to face conversations where I can ask him questions and he can give me answers or he can, you know, actually grab my arm and feel for rigidity or see how I walk across the room, I think those are the kinds of things that I benefit from the most. PwP3 |
| Questions to clarify | - You know, if he doesn't understand, he'll keep asking me questions until he knows what's going on. PwP13 |
| Patient portal | - I see her four times a year, but I can also call her or communicate with her through the computer because she has a portal set up in her office, or in a computer cabinet somewhere, but I can communicate with her that way and it’s helped me tremendously. PwP5 |
| Creating an agenda before appointments helps communication with doctors | - When things are going good, I don’t do it. But when things start to go off, I do start to write them down and keep a schedule and try to find out where the problem is. PwP8 - Usually about a week or two before the doctor's appointment my wife and I will start a list to see what we need to talk about. PwP18 - Yes, and I think some of that has to do I usually go in with a list of my concerns or questions; I don't tend to rely on just my memory because you know we can start talking about a topic and then you know you kind of get off and are someplace else and it's nice to have a list to come back to and say, oh before we go, I just have a couple more questions for you about whatever. And so I try to prepare for every visit, rather than just go in with my memory or whatever. PwP9 |
| Presence of a carepartner | - Or if I'm going to see the doctor, I'm having a particularly bad day, I happen to have a lot of pain with my Parkinson's. And that can definitely cloud your, you know, cloud your judge -- I won't say cloud your judgement, but cloud your memory, your concentration, I guess. You know, so, but his concentration is clear...So if there's something that I'm forgetting or maybe that he feels the answer isn't good enough -- it might be good enough for me, but if it's not good enough for him, he will continue to push. PwP7 |
| Writing down thoughts in advance | - I think that him [my doctor] giving me a chance to write it all down helps a great deal because they give you, you know, time to write it all out and then he will come in and go over each item that I've written down, with me, and ask me about it to make sure he's clearly understanding what I'm talking about. I think that helps greatly. PwP13 |
| Diaries, questionnaires | - When things are going good, I don’t do it. But when things start to go off, I do start to write them down and keep a schedule and try to find out where the problem is. PwP8 - Well I usually bring a couple of the forms of the Davis Phinney Foundation that I fill out before the appointment to try and keep track of what I want to ask him and all that. And they have all of the symptoms of Parkinson's and you know all that. And so I usually give him that or give his nurse that and then he comes in and he spends a good period of time with us PwP6 |
| Videos of confusing symptoms can help physicians identify them | So you know, when I first started getting the dystonic episode, we actually took a video of it in process. So the doctor could see, and again, he knew right away what it was just by looking at the video, so he's -- again, he's very familiar…Again, a video, that's why I think the video was so important because a picture really does speak a thousand words. So he could see the process as it's happening. And because he's so experienced, you know, he knew exactly what was going on. When the first time I had a dystonic episode, everybody was thinking I was having a mini stroke, because the symptoms, you know, look very, very similar. PwP7 |

| **Table 3. Carepartner-reported Barriers to Communication about OFF Time** | |
| --- | --- |
| **Theme** | **Exemplar quotations** |
| Can’t think of a good conversation about OFF time | - I know that they tried to figure out if his … you know, he’s on carbidopa/levodopa and [if] the dosage is right, so they question me a lot as to try to discover how he’s doing, how the symptoms are doing and should there be any medicine adjustments. But I don’t know, I can’t come to mind with a good solid conversation about off periods. CP1 |
| Lack of time spent on/ discussion about symptoms that are difficult/impossible to improve is frustrating to CPs | - it has always struck me that the neurologists seemed to not bother with talking about things they can’t do something [or stuff] about. I tend to bring up you know like the cognitive problems my husband [is doing]. They have no skill for his cognitive issues and they don’t seem particularly interested in dealing with it, and, you know, so that’s my impression is that they don’t talk about things that they can’t fix. CP1 |
| Perceived lack of interest by physicians | - Her doctors have been – again, aloof isn't quite the right word – but we only see them for once every six to nine months, and they're not too keen on like, getting the scoop on this CP2 - You know, they initially said they were going to follow her care. See, we’ve kind of keyed on that word – they're not going to treat her, they're just going to follow her. And that’s what it feels like; they're like following, but it doesn’t feel like an advocacy for us as much as we kind of thought they would be more interested, or something. CP2 |
| Physicians suggesting experience not unique | - I think she’s a good doctor for us, but she said something the other day that – I didn’t ask [name] about it, but it sure bothered me – in our conversation. Just the phrase, “You’re not the only person in the world with Parkinson’s, you know?”… It really pissed me off. Yeah. No, we’re not. CP8 |
| Lack of discussion regarding impact | - If you don’t have that [talking with the patient and get an idea of what’s going on in their life] and you have a patient with a chronic disease that affects all their life, it’s much more difficult to treat the patient well. CP6 - …they kind of pushed early for her to get DBS as well, and it’s almost like they don’t want to talk about it, because the DBS, of course, is supposed to minimise these off-periods. So it’s almost like they – I'm not conspiratorial or anything, but it’s almost like they don’t – They're certainly not asking the level of questions you are, that like, you know, what is the impact and extent and severity of these off-periods. CP2 |
| Technology that shifts attention away from the PwP during clinical visits is a barrier to communication | - I think it’s become tougher with changes in medicine to maintain good communication. So, you know, physician and the patient. And a lot of times it’s the doctor is just forced to sort of sit down and fill out little frames on the computer and not really sit down and talk with the patient and get an idea of what’s going on in their life. I guess, you know, what used to be sort of a holistic approach to medicine and the patient. CP6 |
| CPs see PwP reticence as impacting communication | - …Instead of me not knowing [the extent of symptoms] except just by watching. You know, I don’t know his feelings and stuff, I think if he’d open up more [it would be easier]. CP4 - I tend to be more upfront and let her [the doctor] know what’s going on, where he’s more wait and see, let’s see if it gets worse, when we don’t need to let it get worse, we need to just tell the doctor now. CP5 - Okay, in our lives is the fact that he [PwP5] won’t accept off periods [denies symptoms]. As far as the neurologist, I don’t have any issues [in communicating]. CP5 - He's not a good communicator with his doctor as far as his symptoms. He kind of forgets when he's had a bad symptom, or he'd like to go on to the doctor and talk about golfing instead of Parkinson's CP17 - The biggest challenge is PwP17's honest assessment of his symptoms. CP17 |
| CPs downplay symptoms due to denial/fear | - we have a young lady that we talk with who’s father had Parkinson’s and she kept us abreast of his condition until he couldn’t swallow and died. And so the question is, if you have difficulty swallowing, the answer is, “No, I don’t.” And then we move on. CP8 |
| Lack of CP engagement by doctors is a barrier to communication | - Well, I think the neurologist should enquire a whole lot more of me. I mean, they obviously need to direct themselves towards my husband as a person with Parkinson’s and all of that kind of thing, but I think they miss and have missed a whole bunch of data and information, and it took me a couple of years, not because I’m not assertive or anything, but it was kind of a new experience to be my husband’s spokesperson, but I used to sit there and think they aren’t getting half the story just listening to how my husband perceives what’s happening with him, and I thought it was odd that- I thought it was really odd that the reaction to the person who is with him 24/7 isn’t more important to these neurologists. CP1 |
| Lack of frequent clinical visits are seen as a barrier to communication | - I think the neurologist is really, really good, but by definition that means he's really, really busy and I probably would like more frequent communication whereas it can be six to eight months between visits, and she's currently has - will call in the middle of that to change a dosage or something like that, but for the most part the frequency doesn't seem to be there. CP7 |

| **Table 4. Carepartner-reported Facilitators of Communication Regarding OFF Time** | |
| --- | --- |
| **Theme** | **Exemplar quotation** |
| Importance of listening | - It certainly would help for the physician to ask open-ended questions and be patient to listen to the responses, that would help. CP1 - She actually sits and listens, and she doesn’t try to make you think that something else is happening CP5 - Just the fact that he takes the time to listen and has worked with us to find solutions that will work for my husband. CP12 |
| Listening to caregiver | - You know, the doctors definitely are open to talking to both of us and, you know, I certainly get the feeling that he’s as interested in what I observe as what my husband observes. CP12 - He asked me, you know, how I felt [patient name] was doing and was totally open to listening to me. CP17 |
| Asks about daily life | - He sits down, you know, and talks and how are things going and blah, blah, blah and what are the children doing and all this – and the grandkids. And, you know, really gets the idea rather than just say, come in and sort of just bop, bop, bop, by the book type little questions. CP6 |
| Relaxed, eye contact | - He sits down and he’s relaxed. You’re relaxed. He looks you in the eye and he goes back and forth and talks. You know, you carry on a conversation. You come across as interested as interested in the patient. CP6 |
| Appreciates individuality of experiences | - you know like she doesn’t say well, no, that can’t be, you know, even … because everybody is different and some things affect people in different ways, so she never says, well, you know, that’s not possible, kind of thing. CP5 |
| Separating data entry from the clinical interview benefits communication | - Most of the time he will sit there and instead of trying to enter data simultaneously as he talks on the computer, he will go through and talk and put this stuff down. And then he’ll sit down and say, “Okay, let me enter this stuff into the computer,” after he gets through, you know, the meat of the clinical engagement, which I think is probably the best thing he can do. CP6 |
| Physician addressing specific concerns | - Just that I think that our doctor has been particular good about addressing what we were uncomfortable with, well especially my husband, and I’m very pleased with that because you know another doctor might have just said, you know, “Too bad, this is how it works”, you know and this guy really went the – you know, has really worked with us to try to find a solution that is beneficial and I think we have. CP12 |
| Physician providing future options | - Well like for example the new neurologist, you know, just told him that you know while he’s not a candidate yet, you know, there is brain surgery that he can have that can be helpful with this, and we had looked into the brain surgery and so we’re not quite there yet. But it was an – you know, it was some type of positive prospect instead of, you know, the original first neurologist just seemed to have the – give the opinion that you know, “You’re just on a constant downhill slide and it’s going to get worse and worse”. CP19 |
| Patient being open with experiences when talking to physician | - I think she's very upfront and complete with her communication with the doctors... I think she shares very openly whatever she's experiencing so I think that's a positive aspect of her… CP14 |
| CPs help communicate extent of symptoms to physicians, when PwP do not | Carepartners help when patients are innacurate   - So I go to all doctors’ appointments with him, every one of them, because he doesn’t remember things, or he’ll … not intentionally, but he’ll forget to tell things…CP5 - Dr. X asked her yesterday about what percentage of the day did she have dyskinesia. And Candy said, “Well, not much. Maybe 10%.” I had to say, “That’s wrong.” I wish it was, but I would say minimally 40%, 50% of the day. CP8   Carepartners help when patients downplay symptoms   - I try to tell him, the doctor, more than he does. He tries to put on a good show and make him look, he wants to look the best he can… So he works extra hard when we’re at the doctor to, you know, walk up straight and all of this. I tend to bring up more of his problems than he does. CP4 |
| CPs help absorb information given at clinic visits | - Now we’ve learned that for either of us it’s necessary for the partner to be in the room with the doctor because you don’t hear or, you know. Especially when there’s bad news [e.g., at her diagnosis of PD] and all of a sudden you go deaf. CP8 |
| CPs appreciate alternate modes of communication in between clinical visits as needed | - She does have a patient portal where I’ve communicated with her [for urgent matters]. CP5 - He sees his neurologist twice a year, every six months, and has access to patient portal, has access to email, has access to phone. If there were a crisis he would have no trouble getting in touch. CP18 |
| Keeping an agenda for clinical visits helps | - Well, we both will make notes if something unusual happens and keep a list of those things and then go over them with her so that we’re prepared. I think preparation is the best thing. CP5 - PwP18 goes to his appointments with his binder, he has a Parkinson's binder, where he keeps track of his exercise and his medication and he writes down questions. I write down questions. I mean, I don't think you can just show up in the doctor's office and expect them to read your mind. You have to be able to tell 'em what you're concerned about, what you're thinking about, what's going on. CP18 |
| Questionnaire that is completed by CP and PwP together may help stimulate discussion/improve communication between them (suggestion from CP) | - I think for us [a joint questionnaire] would be a jumping off place to – for the two of us to talk about it a little bit more without it being, you know, depressingly uncomfortable. CP8 |
| CPs wonder if using videos (on phones or tablets) to demonstrate off periods, but are concerned about invading privacy of PwP | - I do have a tablet. And I wonder – but I think it would make PwP8 feel weird if I videoed it and shared it with Dr. X and say, “Well, this is,” but I know – I’m pretty sure I would know how she would feel about that… Invaded – if I was recording unpleasant moments. I may be wrong. It’s probably something I should talk with her about. CP8 |

| **Table 5. General Neurologist-reported Barriers to Communication about OFF times** | |
| --- | --- |
| Theme | Exemplar quotation |
| Lack of patient insight | A lack of patient insight or ability to sufficiently express it would be a barrier [to communication] GN3 |
| Cognitive impairment is a barrier | **Cognitive impairment makes it hard to determine the problem**  If the patients themselves are having, has got concurrent cognitive problems, that makes kind of their understanding of what's happening to them in their body more difficult. GN2  Sometimes the patients with really bad cognitive problems it’s really hard to tease out is it an off period, is it the disease, is it something else, you know is it arthritis or something. A lot of times it kind of depends on the historian, if someone has cognitive issues related to the Parkinson’s or something else then it’s pretty hard to keep track GN4  Other challenges might include patients who have some cognitive impairment. And so they may not word things as well. They may not be as prone to -- either spontaneously or on direct questioning -- endorse or be able to explain their symptoms well GN8  And, if they have any, you know, cognitive impairment, it only makes it more challenging to associate any of the symptomatology with timing of the medication… this person is also 80 years old and they're having a hard time, you know, trying to explain it and trying to find out when they last took their pills. And, they don't remember. And, they don’t know which one. GN10  **Cognitive impairment makes it hard for patients to understand explanations**  And it becomes particularly more difficult explaining all that as dealing with patients who have more cognitive symptoms GN2 |
| Lack of historian | Some patients who are further along may be in a facility, perhaps assisted living or skilled nurse facility, and thus may not be accompanied by appointments with family or a significant other. They may show up dropped off by a Medi-Cab or some other transport by themselves. Or they may arrive with a -- you know, a transport personnel or home health type personnel from the facility, who is generally someone who brings a binder but is not necessarily aware of the patient's disease, meds, or issues, and doesn't -- can't add anything of value. GN8 |
| Caregiver’s cognitive impairment | Similarly if a caregiver's physically older and having some cognitive problems themselves trying to explain that [off periods] can be challenging. GN2  Another issue is that many times you have an elderly couple, so you have two patients and one may be the caregiver, they may both be caregivers for each other for different things GN1 |
| Differing accounts from PwP and CP | You're running up against the difficulty of talking to someone and someone…and the patient saying one thing and the family member saying something else. And, there could be some disagreement and argument about it. GN6  once in a while, you have a patient who's willing to be treated and has fluctuations recognised by others but has kind of a denial or a limited acceptance of the diagnosis. That is the only other category I could think of. You know, their families will then say, "No, no, no. You know, they clearly, you know, do great for four hours, and then we've got to, you know, move them along or coax them into the next dose, et cetera." And the patient's like, "I just don't have a problem." GN8 |
| Memory/recall of symptoms | Well, on one hand you're relying on people's memories of what they're experiencing. They may not be able to accurately say how their symptoms relate to the timing of their dose. GN7  [Another barrier] also may be just a recall bias [in OFF symptoms reported]. GN1 |
| Difficulty linking symptoms to dose timing | They may not be able to accurately say how their symptoms relate to the timing of their dose. GN7 |
| Lack of shared vocabulary | I think one of the tough things that makes it tough to communicate with them is not speaking the same language when you're trying to…not like, English, not another language, just when I'm asking a question, if they don't understand what I'm trying to get at. GN6 |
| Non-English speakers | I have a handful of patients with Parkinson's who don't speak English as a primary language, and a family member translates most often – [this is a barrier to communication] GN8 |
| Poor PwP/CP understanding of symptoms | For example I've had patients who actually who think that dyskinesias, a manifestation of the Parkinson's itself [and therefore an OFF symptom]. GN2  Well sometimes it’s hard to tease it out. You know, sometimes a person is not really aware what an off symptom is, sometimes it just gets filed under, “Oh this is my Parkinson’s disease”. GN4 |
| Clarifying fluctuating symptoms | You know, patients just say they don't feel well all day long. And, so, this is hard to kind of say, "Well, was there a time when it was really different? And, how does it relate to my doses? Is it the beginning of my dose? The end of my dose? And kind of arbitrary?" You know, they just feel badly all day, so it's hard for them to pick these out. GN7  So, it's very difficult for us to determine that. Is the tremor worse because of an off phenomena? Or, is it because they're taking an extra dose or whatever…too much in the evening or bedtime dose, now they're getting the dyskinesias that are due to too much Parkinson's medicine. GN 10 |
| Time constraint | Time constraint - in Parkinson’s is there’s so many things you kind of need to ask about and although you try to screen for this every time or at least when you feel like you need to screen, there’s a lot of other things you just kind of got to get through. So certainly that can factor in [as a barrier]. GN3  The diary, I do have access to that. But, it's a time constraint. GN5 |
| Lack of ancillary team support | Yeah, I mean where I'm at, I'm in a real small practice, it’s just myself. GN3 |
| Having too much data | I don’t know how to say this, I feel like diaries and Parkinson’s are probably a useful research tool over short terms when evaluating specific interventions over like, you know, over a four, six month time frame between visits. I think it would be just an almost overwhelming amount of data to evaluate. I think it would be like the equivalent of if an endocrinologists asked for their patients last six months of finger sticks instead of getting an A1C. GN1 |

| **Table 6. General Neurologist-reported Facilitators of Communication about OFF time** | |
| --- | --- |
| Theme | Exemplar quotation |
| Making an effort to listen to PwP/CP in clinic visits | I think trying to be cognizant of not being rushed when I'm actually in there and when I see a patient I actually don’t, even though I have an electronic medical record, I actually don’t type or work on EMR when I'm seeing a patient. So, trying to be present with them and having a conversation with them versus trying to do my data entry at the same time I'm talking to them. GN2  Well, I think the main thing is just, you know, probing, asking questions, giving people an opportunity to say they're having problems. GN7 |
| Long term relationship helps communication | As neurologists we’re their primary care doctors of their Parkinson’s disease and so, you know it’s a long term therapeutic relationship and so the pragmatics of that is one does not have to feel that they have to shovel everything into a given visit. GN1 |
| Asking consistent questions | Querying for these symptoms in a consistent, repetitive fashion gets the… And in a way, that, you know, is understandable to that patient, or, you know, kind of just gets them used to the questions and used to kind of self-monitoring. GN8 |
| Understanding individual preferences | To some people more dyskinesias might be more acceptable. For other people more, not necessarily being completely off but being kind of more on that down slope of motor symptoms maybe where they're more comfortable based on kind of the activities that they do. I think it's important to ask about that 'cause I think sometimes we get so focused in clinic on treating what we think is the priority or what level of function we think that they should have that sometimes I think we forget to ask them, to ask our patients and their families, what's most important and what they value. GN2 |
| CPs provide collateral information | The caregiver supplemental history especially if the primary patients history is let’s say vague or unverifiable [is helpful]. GN1  It also helps I think when a patient comes in with someone who’s an observer of them as well, because sometimes they might not have the best insight, so it certainly helps if I have another family member or close person who says “No, I noticed that after this many hours” or whatever. GN3  I still feel like the presence of the caregiver is extremely important in these situations and very similar to patients with Alzheimer's disease, you know, to get a collateral history right. GN5  Because the caregiver is very central in treating off periods sometimes. Especially if you get more severe into the disease. GN9  "Is this is something we can talk about amongst everybody here? Do you mind if we hear from family members so we can get a wide range picture of what's going on. And, you know, sometimes you have a different perspective than your family member does." And, for the most part I don't think there's really been any cases where patients have said, "No, I don't want this person who is here with me to speak in this moment." So, it kind of becomes a joint conversation that we have. GN6 |
| Home videos to clarify symptoms | In this day and age of the video, you know, if they have their phones and they have video, they might be able to record what they're talking about. So I have an idea, you know, if it's something I am unable to see or witness at the time that they're trying to communicate it GN10  Maybe the [home] videos too, every now and then, if something is a bit confusing when it's described. GN5 |
| Motor diaries | And I think it would be helpful because it would allow patients and their families I think to be more engaged. GN4  you know, a clinic visit is a snapshot in time. I'm counting on them to be able to remember all the things that I'm asking them, which can be a lot. And they may forget important details so, you know, diaries would be helpful in including [mobile] diaries that they can do this while they're at home so then it's not them trying to recall depending on the last time I saw them three months, six months of information when they might forget. GN4 |
| App | if there was an App that was really well-designed. Not for the patient themselves, but, like, one that’s geared for family members to monitor and track. Maybe that would be helpful.GN6 |
| Questionnaires | It may be potentially helpful to have some of that work where they would complete like a scale or worksheet or some type of not particularly complicated but then that would prompt some discussion during - to help streamline the actual interview if there's limited time with the patient. GN4 |
| education by physician | the best [educational tool] is really counselling and explaining GN9 |
| Teach-back method | But, another one that we use quite frequently…"Tell me what I just said to you. Like, let's discuss what I just said, so that way I can hear that you're understanding what I'm saying." GN6 |
| Allied health are seen as helpful | [The benefit of allied health available would be] … the education. I mean, instead of me going through that period of explaining things to them and talking them through it, it would be them doing that. GN9 |
| Using basic graphics to demonstrate OFF periods relation to medication timing | I'll draw graphs of kind of what a typical or an off period may look like and how medication fits in there. And kind of I'll hand draw those with some Parkinson's patients. GN2 |
| Using video educational tool regarding OFF periods | very short narrated video clips of different motor phenomena, you know, during the off period, very clearly narrated. I think that would be very helpful to patients. Maybe more so to caregivers, especially if the patient is an advanced stage. GN5  I tend to use the Michael J. Fox in his public service announcement as an example, when he was campaigning for the Missouri Senator, with [unintelligible 00:08:05] exacerbating his symptoms, or not taking medication. Let me show you this video. This is him having dyskinesia as opposed to tremor, versus some other movement disorder.GN6 |
| education by online forums or community groups | I offer my patients to join forums. There's local classes where they explain a lot of things Gn9 |
| Pamphlet unlikely to be helpful | A pamphlet wouldn't be very helpful to show abnormal movement GN5 |

| **Table 7. Movement Specialist-Reported Barriers to Communication about OFF Times** | |
| --- | --- |
| Theme | Exemplar quotation |
| Distinguishing tremor (OFF) versus dyskinesia (ON) | "And patients are not very good informants about these kinds of things and I've had several experiences where what patients describe – thought was – they thought were describing worsening of their motor function, i.e. a tremor during an off period, actually turned out to be peak dose dyskinesia or the on/off dyskinesia...that can be very confusing and make it very difficult to adjust their regimens" MDS1  "...distinguishing between, say, a dyskinesia and a tremor can be very difficult for a partner or caregiver."MDS2  "And shake is very vague because shake could mean dyskinesia or it can mean tremor ." MDS3  "...dyskinesia is just really hard to, for some people, to distinguish from tremor despite explaining it multiple times." MDS4  "Sometimes the misperception about dyskinesia and what is tremor and what is dyskinesia, can sometimes make – well, that’s just a challenge that is inherent in the conversation, that many patients don’t understand the difference between tremor and dyskinesia." MDS7  "A lot times patients misinterpret their dyskinesia as tremor and that can be confusing because when they’re really on they think they’re off and they’re worried about that movement when it’s really an on phenomenon" MDS10 |
| Unclear if dystonia is off or on phenomenon | "Sometimes dystonia is when they're on and sometimes it's when they're off, so that can be really challenging from my end to interpret. Cause sometimes they have that even, you know, before they start with Levadopa, it's just as a manifestation of the disease, and then it's unclear whether it's better or worse, or, you know, part of the dyskinesia, so that is, that can be a big challenge actually, in terms of understanding the temporal relation." MDS5 |
| Dyskinesia harder to understand than off time | "They seem to have it pretty down on versus off explanation but dyskinesia still tends to me an issue for most people, even those people educated. Sometimes it's hard for them to assess whether are they even dyskinetic, is that dyskinesia actually in a Parkinson's symptom or med related symptom? Is that dyskinesia troublesome or not? " MDS9 |
| Non-motor fluctuations more difficult to recognize | "People don't always make the connection between some – particularly between some of their behavioural manifestations and, you know, their fluctuations in their medication schedule over the course of the day and they're not always good about putting those two things together and so that can lead to a delay in recognition that they're having some kind of non-motor problem which is related to their medication schedule." MDS1  "think the other challenge is when somebody has non-motor manifestations, like anxiety or nausea or, you know, you know, mental issues, that kind of -- like, cognitive slowing -- and sometimes those can be more subtle because anxiety and cognitive issues can be unrelated to Levadopa and can persist even when somebody's perfectly on. So, that can be challenging to differentiate." MDS5  "Plus, the mentioning, not only the motor symptoms that can worsen but also the non-motor symptoms that can emerge during an off period." MDS6  "The other thing is that so much of the time the off-periods are – so there are psychological symptoms, and if they're associated with obvious severe motor symptoms, then that’s not hard to ferret out. But so many patients don’t understand that the motor wearing off might be mild and the emotional symptoms might be much more severe." MDS7  "I think the people who are the most difficult in clinic are the ones who have a lot of non-motor off, especially anxious patients I think can be very difficult to get to the bottom of the on/off and separate the anxiety from the off" MDS8  "I think the people who are the most difficult in clinic are the ones who have a lot of non-motor off, especially anxious patients I think can be very difficult to get to the bottom of the on/off and separate the anxiety from the off" MDS8  **Uncertainty regarding how to treat non-motor fluctuations**  "I never know what to do with non-motor fluctuations unless they are accompanied by motor fluctuations quite honestly. I always go for that first and then I try to fill in what the rest of the experience of off is for them. Because I don’t know how to treat that. I just don’t know how to treat that even for medicine for these non-motor symptoms that I know of that I can, at first I have to put it in the context of motor. That’s just me I guess." MDS8 |
| Anxiety can impact experience | "But, you know, that's one of the issues with anxiety is that there's a lot of anticipation, you know? With anxiety, it's that oh my god, I'm going to be off, and I took my medication, I woke up late, da, da, da, you know. There's a lot of that going on with a lot of these anxious patients, but I think that it's really a matter of insight with some of the patients." MDS5  "I think the people who are the most difficult in clinic are the ones who have a lot of non-motor off, especially anxious patients I think can be very difficult to get to the bottom of the on/off and separate the anxiety from the off..." MDS8 |
| Identifying whether or not issue is a fluctuation | "So one of the challenges is that if a patient is doing poorly that it could be because of a true off phenomenon or it just might be a symptom that doesn't fluctuate at all." MDS2  "sometimes people describe this sort of very vague, you know, I just don't feel good in the morning. [laughs] Or, I just, after lunch I'm this, you know, I need to take a nap and I feel so tired. Or I feel so fatigued, or I feel so weak. So, that's obviously a bit more challenging, cause I mean, who knows? They could be having orthostatic hypertension, right? They could be, I don't know, just sleeping, having sleep apnea, sleeping crappy and feeling tired for that reason. So, often times it is a bit more challenging to really find out what's going on if they, I mean, you know, some patients or sometimes it's very straightforward, they're just saying oh, you know, half an hour before I take my next dose the tremor comes back. You know, so, well okay, I'm kind of done, you know? I know what's going on. But, but, but again, I think mostly when people describe their off, or their possible [laughs] off symptoms as very vague, it makes it much more challenging." MDS4  "The big thing is understanding what is related to meds, and I think that's a challenge on my end, too, because you know, sometimes patients can have good days and bad days, and you know, or sometimes medications can be really sporadic where it's not really clear-cut in terms of what's going to work and what's not going to work." MDS5  "I think the biggest challenge is when patients don't reflect their fluctuating symptoms to changes in meds." MDS5  "And freezing as well is another example of a non-motor -- is an example of a symptom that's not always Levadopa-responsive. So, sorting out whether it is or isn't is the challenge of is it off or not related to off? I think that's the big one." MDS5  "But there’s some peculiar symptoms, you know, people can, can complain of a lot of pain during the off period but you want to make sure that, you know, when you’re, when you’re assessing these non-motor symptoms that it’s not something else, in other words some other symptomology other than the wearing off." MDS6 |
| Trouble describing what they are experiencing | Like I said, some of them are really clear, like they know their body's really well and some of them are, you know, unclear but then it's not because of the denial but rather because they have a hard time really being exact about what they're experiencing." MDS5  "Some people, it's crystal clear to them. You know, they feel it in their body, they know exactly what it means to be on or off, they know what their body's supposed to feel like. And then some people maybe, you know, I guess the way I would put it is it's one of those unknown unknowns." MDS5 |
| Cognitive impairment | "One of the problems with on-off and communicating is in terms of their cognition ability. If they are having cognitive problems, then – then it’s of course harder to communicate." MDS7 |
| Disagreement between patient and caregiver | "Yeah, usually I’ll ask the patient and I’ll just say you know this person [the CP] is disagreeing, what do you think about that? Is there any truth to that and sometimes that changes their answer and then if it’s a patient that, like we do basic mental status testing on all of our patients and so if it’s someone who’s done poorly on their mental status testing then we would tend to agree with the caregiver." MDS10  "Patient and caregivers disagreeing on what they’re feeling can be challenging" MDS10 |
| Variability of symptoms between individuals | "I'd say the variability, all symptoms vary quite substantially between individuals, so it's not sort of where I can say, here's a list of things, you know, more tremor or feeling slower or stiffer or things like that, because I think that people experience off differently but also some of the features of disease they experience, and so what - you know, as we mentioned before, what is disease progression versus just, you know, and lack of peak dose efficacy versus off . " MDS2  "Sometimes dystonia is when they're on and sometimes it's when they're off, so that can be really challenging from my end to interpret. Cause sometimes they have that even, you know, before they start with Levadopa, it's just as a manifestation of the disease, and then it's unclear whether it's better or worse, or, you know, part of the dyskinesia, so that is, that can be a big challenge actually, in terms of understanding the temporal relation."  "[They are] different from patient to patient, some patients being much – having much milder symptoms and other patients having severe symptoms. Some patients having severe motor symptoms and some patients having severe psychological symptoms, and some patients having both. And then some patients in the earlier stages might have mild motor symptoms that I can see, but feel very impaired. So it’s really variable – so variable form patient to patient." MDS7  "You know, this condition is so tailored differently from patient to patient that I think that the definition is a little different from patient to patient. " MDS9 |
| Patients not admitting that they don’t understand what physician is saying | "I think one thing is that because physicians are so highly trained they forget how untrained people think and since patients will kind of go along with their doctor, they may kind of indicate that they know what the doctor’s talking about when in fact they don’t. So I think that is a major, I’m sure that happens in my clinic, they’re nodding their heads and walk out of the clinic and one turns to other and says what the hell is she talking about." MDS8 |
| Doctors and patients have different expectations | "I mean first of all this is not common lay terms, right? These are all terms that movement disorder neurologists or general neurologists have come up with to help better describe some of these states with Parkinson's disease. But this not common verbiage for the lay folks, you know, and they hear about it but they don’t quite understand it. I think the big issue here is that even though you get 75% improvement, you know, from taking Levodopa for your Parkinson's symptoms, maybe patients fixate on the remaining 25%. So, when you talk about an off state, an off state in their mind may need, you know, a place in which my symptoms are prevalent. Whereas an on state is complete resolution of all of my symptoms coming from the Parkinson's disease and that's I think sometimes their initial approach that I want all my symptoms to go away. Well, if you get a Levodopa response at 25%, you know, the medical field, that's great. That's amazing, that's an on state until proven otherwise.   But maybe to the patient it's not. So, I think that patients maybe come in with a different expectation, different level of understanding and you sometimes have to just realign that again and that's where the difficulty is." MDS9 |
| Lack of shared vocabulary | "I think sometimes the patient has different descriptions for what they’re feeling then for what we ask them. So just wording can be challenging." MDS10 |
| Recall | "...recollection bias might also be a -- I mean, I think ideally if they had sensors or you know, really give us a more accurate picture than probably the patient's recollection, so. That might also kind of be a factor." MDS4  "I would also say recall bias, and I think that's why, you know -- and it happens to all of us, you know, how many times did you have a headache in the last two weeks? Well, you know, you can't always remember very clearly, so keeping a diary is helpful in those patients as well." MDS5 |
| Time *not* as much of a barrier for movement disorder specialists | "Not particularly about off. The answer is yes, but that's mainly just a time constraint in discussing Parkinson's and managing Parkinson's in the allotted period ." MDS2  "No [time constraint is not a barrier], because it's the first step after we reviewed the medication that's usually - I dive into asking about on/off." MDS3  "Well, I'm kind of fortunate that I do practice in an academic setting, so I mean, that's usually not an issue for me, I would say." MDS4  "I don't think it's time constraints because oftentimes, that's the main topic of discussion. So, it would be something I would focus on, you know, and I don't think time is an issue in that regard." MDS5  "Time constraints – for me, I always focus my efforts on psychological issues, because I just think that that affects the quality of life more than the motor symptoms, and so, I don’t have a time constraint in that regard. " MDS7 |

| **Table 8. Movement Specialist-reported Facilitators of Communication about OFF Periods** | |
| --- | --- |
| Theme | Exemplar quotation |
| Including CPs in discussions to improve OFF communication | "I think definitely including the family, or you know, whoever lives with the patient in that kind of discussion [about OFF], I think is really important" MDS4 |
| Levodopa challenge (to understand and to educate) | "I will say is doing the Levodopa challenge, which I don't do consistently for the intestinal gel patients, but certainly for the DBS patients, and I think that also gives the patients a perspective on what is off and on. It's a little less - it's certainly a little bit more artificial when patients come in off in the morning having offs during the day but I think it helps illustrate the point as well." MDS2  "I have maybe one patient per month, they're kind of just feel, I'm not entirely clear is what -- you know, very, they just are patients who will call you and say, you know, what we're doing is not working, I just feel bad, I feel weak, [laughs] I feel the medicine isn't working and I'm not really quite sure what's going on. So, yeah, so that's kind of the situations I would bring them [in for levodopa challenge for OFF communication]." MDS4  "...these studies, they always – the ones that are measuring off-times, they always have a diary that the patient takes home. And in the clinic, there, we have two to four hours at one of the first visits, where we do concordance testing, where the doc and the patient have to agree on what state the patient is in every half hour, over two to four hours [i.e., levodopa challenge]. And so that’s educational for the patient, if they are thinking that their dyskinesia is actually tremor, or vice versa." MDS7  "But I only do that [levodopa challenge] in the patients who I'm not quite certain about what their off periods look like. They are not quite certain and they can't really express that to me so then I'll bring them in in an off state. Typically I want them in in an on state but yeah, but I will then, in those people who I'm not really getting a clear answer from them or they have no family around and I'm not getting that information I need, I'll bring them in in an off state." |
| Developing shared vocabulary | "And also, what I do is, I sit down with the patient and I have just a blank sheet of paper and big lettering, and I put what’s on in one column and what’s off in another column. And they’ll tell me, okay – I’ll say, when your medicine is not working, what do you like? You know, if they do have tremor, and so they’ll say – right off the bat they’ll say something, and so I write that down.  And they might say, I can't walk – they say I'm fine, I'm sitting there at my desk and I'm getting a lot done, but then I stand up and my feet won’t go, and that’s why I know, oh darn it, I missed my dose two hours ago, and that was the only thing – they didn’t even know until they stood up.  So then I write down, okay, your feet won't move, then I use their terminology and I write that down in the off column. And so then they take that piece of paper home and when they're filling out their diary, then they can look and say, now which one do I fit – the on or the off column – right now? So that’s another tool." MDS7  "...the reason that that [running through medication timing and OFF symptoms] helps is that I can understand the language that they’re using and try to think it up with the language I’m using. I view it as an educational opportunity experience basically." MDS8  "...I give them kind of language because I think the most important thing is a shared language and a lot of, you know, a lot of times a person will use tremor as their main sign of off which isn’t a particularly a good one, so kind of talk about what the more important features are that really define the difference between on and off." MDS8  "The most recent difficult patient was a psychiatrist in fact who’s got terrible anxiety and also definitely fluctuations and so she had, her diaries had three different words. One of them was gluey... which it turns out that that probably is freezing...So anyways she had gluey which meant freezing and then she had, what was another one, I can’t remember what it was but it was another word that seemed to be more with the off so we had to spend a long time talking about what gluey meant to her and it really meant that her feet were stuck to the floor and what that other word was, I’m trying to recall, but it was very helpful because once we equated that off state that she was experiencing and gluey was this freezing and I had a much better idea about what the relationship of her fluctuations were to her medication schedule. She’s doing a lot better, but you know, it’s that kind of a thing. " MDS8 |
| Motor/symptom diaries as tool  (dissenting view: too labor intensive) | "I may send them home with diaries if I’m having trouble understanding…" MDS8  "Have them define the word and if there’s a care partner there, what does that look like to you and try and send out another set of diaries and see if it makes more sense. Diaries are cheap." MDS8  "And I think that, you know, using a diary can help, especially if you sort of get into, you know, how do you feel when you're on, and how do you feel when you feel like not good, I guess is the other way of putting it. It's funny, cause people use different words for it [OFF]." MDS5  "If they're fairly clear about what they're experiencing, and you know, they have a clear-cut sort of fluctuation that they can identify without necessarily needing to get into the nitty gritty of the diary." MDS5  "I think mostly when people describe their off, or their possible off symptoms as very vague, it makes it much more challenging . And then in these cases, I might either again, based on the ability, the understanding of the patient, I might either give them like a motor diary home to fill out" MDS4  **Dissenting view: diaries are too labor-intensive**  "From a practical point of view on the rest of our patients, the studies – I'm sorry – the diaries are pretty labour-intensive, and we don’t - used to in my early years, send a diary home with a patient and ask them to fill it out for about three days in the couple of weeks before they come in. But one day will be so different from another day, that you may – it’s like generally – sometimes about 50% of the time – that was useful and overweighed the trouble, and the 50% of the time the patient wouldn’t do it, or it just wasn’t useful, so I stopped doing it eventually. " MDS7  "I used to use motor diaries. I find that I go - it takes way too long for me to explain how to actually do a motor diary. " MDS9 |
| Wearable technology as tool to clarify OFF communication when needed (2 MDS, MDS9 and 10) | "And then there are few instances where I actually have them wear wearable technology in order for me to assess that objectively in the home settings." MDS9  "there's a lot of devices that are out there from various companies across the world that resemble, you know, a Fitbit or a glorified calorimeter. They basically strap this to their wrist and it picks up information regarding their level of function, the mobility, their sort of dyskinesia and their tremor and relative to when they took their meds. So, now you can graph out what their med response is, what time they're sort of in their better state, do they actually wear done and off and does the next dose get them back to where they were before? So, sometimes that gives me a much better look objectively on how they're doing over a longer time period because you can sometimes record from this for nearly a week. Whereas when I'm seeing them in the clinic for a 20 or 30 visit which by nowhere is a reflection of what's been happening at home." MDS9  "It used to be kind of hit or miss. If I see somebody whom I'm not really getting a clear description about on versus off time and I really want to assess whether the meds are even effective or not I'll do it. But I may have to - 'cause that's somewhat, you know, haphazard, there's really no rhyme or reason to it other than just patients coming in. And that makes me so uncomfortable that I'm only capturing patients if they happen to come in.  So, I feel like that's a very crisis driven model that, you know, only when patients come in and only when they report to you or lack of reporting to you am I actually sort of deploying this particular method. So, I think it might be better for me to find other ways in which I can actually kind of broaden who I send this technology to. Ultimately it would be great to get it on everybody but, you know, I'm not sure that my clinic has a bandwidth for that." MDS9  "Yeah, the Fitbit like product gets mailed out a patient. When they complete using it, they mail it back to us, it's an envelope that's already pre-stamped, you know, prepaid. And then we upload it to a system that's secure and then that data is basically transferred on a secure email system." MDS9  "Yeah so the motor diaries, usually you're doing it over three consecutive days and you're usually assessing whether a patients off, no, with troublesome dyskinesia on with non troublesome dyskinesia or just plain asleep. And so it gives you some idea as to pattern relative to taking the medication as long as the reporting is accurate, as long as the reporting is consistent and the reporting is done with a clear understanding of what these things are. On the other hand, the Fitbit like model, what it's doing is it's just capturing data. There's no need to - it is really for the provider to assess is the patient in an on state is the patient off state how is this relative to the medication? Is the patient very dyskinetic? So, I find that that to me is a more helpful tool that is consistent as oppose to the motor diaries that like I said just relied entirely on the patient giving you this information or understanding the information."  "If we have someone that we are suspecting a lot of motor fluctuations then we use PKG devices which are like personal kenosis-grams they can wear on their wrists for a week and it will give us a report about percentage of time that they looked to be off, which the device interprets as when they’re really bradykinetic" MDS10  "We have found it [wearable PKG technology] beneficial in patients who are not able to reliably explain the timing of their on and off periods and it sometimes will give us a better picture of what times of day they actually really do appear to be off and a lot of times it’s different than what the patient is telling you. But when we make medication adjustment based on that, like those results, we’ve had kind of varying results in how successful that is." MDS10 |
| Physician education using analogy | "We also use the analogy of a roller coaster with the patients and saying that there’s up and downs and trying to describe that the medication can have like a peak affect where they feel good and might have side effects and then it starts to come down and eventually they will come back to where they have all their symptoms back and I think you just use as many analogies like that has been helpful" MDS10 |
| Hand-drawn graphics | "I usually kind of start drawing kind of my own little graphics to kind of explain like, oh, you know, when the Levodopa peaks and then it wears off, and then, so I mean, I suppose you could have some more like standardized materials for people to give on this. Maybe specifically on motor fluctuations." MDS4  "Sometimes at the beginning or somewhere along the line, I'll draw a little, a little diagram for them, you know, where it's like being in the right window versus having too much and then having dyskinesias and then too little and feeling off. " MDS5 |
| Desirable: handout | "But as they leave it would be actually quite helpful to have something to hand to them something that's written in simple words with no graphs. You know - where I'm used to turn[ing] the screen and show[ing] them a pharmacokinetic graph of on/offs, you know, fluctuations of Levodopa, it's way beyond their heads, I've realized. So I don't do that anymore. But something simple, maybe like even a card, or you know, like a one-page, you know, like a foldable one-page sheet that not only explains on/off fluctuations but also explains dyskinesia, you know, and how to use terminology. Things like that. I'd see a role for that. " MDS3  "Sometimes at the beginning or somewhere along the line, I'll draw a little, a little diagram for them, you know, where it's like being in the right window versus having too much and then having dyskinesias and then too little and feeling off. " MDS5  Conflicting view: |
| Educational tool for non-motor OFF symptoms | "I think some of these non-motor things, particularly the anxiety disorders which can be associated with off periods that, you know, having some education about that would be helpful, might also avoid multiple emergency room visits." MDS1 |
| Concise video tool | "It would be an interesting experiment to try and develop like a set of videos, a very concise set of videos for [communicating about OFF]." MDS1  "I've considered doing... is showing a video of a patient that's...on medication and then off medication, and I feel that can be beneficial, but I find that just to describe the - the fluctuation for patients who just may be experiencing some mild degree of fluctuation I think can be counterproductive because often those are very exaggerated examples of somebody who is very impaired, and I don't want them to become - you know, disheartened that this is their potential future, because that's not necessarily a guaranteed thing. " MDS2  "I think the idea of a video is nice because you can show okay, this is what this person looks like when he's on, and this is what he looks like when he's off. But again, sometimes there are these non-motor manifestations, so I'm not -- you know, I think that that would be something that would also need to be discussed." MDS5  "I think the other thing that would be helpful in the video would be dyskinesias versus tremor. " MDS5  "...when we have these patients enrolled in studies, then they – they often have this videotape that they watch, that shows patients having dyskinesias, and a patient being on and on with dyskinesias and off, so they can see the three different states. And then we make sure that they understand that." MDS7  "I was just at an investigator meeting and they had the Bob Hauser dyskinesia diary rating tape, the training tape that a patient watches and that’s a pretty darn good resource. I don’t know that that’s available to patients on the web." MDS8  "Sometimes I'll direct them to, you know, like videos like, you know, Robert Hauser's original video on the differences between these things, those in particular for our study patients" MDS9  **Caveat with using video education:**  "But the downside of those videos are that when we use them in the motor fluctuation studies, when patients at least have had the disease for long enough to have developed on-off, you know, we don’t want to show that video to a newly-diagnosed patient, because it will make them – I'm concerned. Even in my more advanced patients, sometimes when I’ve shown it, the patients have gotten upset, because to make it clear, the video shows fairly dramatic patients, so that kind of upsets them to see how severe off could be, how severe dyskinesia could be." MDS7 |
| Interdisciplinary team, advance practice providers  These quotes also note that APPs lead education programs in several clinics | "I think more people discussing it, people having a little bit more of a - you know, separating out some more qualitative and quantitative discussions, but I think more a repetition, you know, based thing as saying, all right, this is an off and having somebody that can explicitly train...So in the same way, having kind of an educator, whether it's a nurse educator or somebody else on the team, I think absolutely can be beneficial. " MDS2  "So, we don't have a nurse, but we have a nurse practitioner. She literally just joined us last week....I think [an interdisciplinary team] could be something that, you know, would help in terms of education if the. . . You know, cause sometimes, you know, I'll admit it, sometimes, like I said, sometimes the physician is like, oh, I did not realize that this person did not know what I was talking about, and they're [laughs] sort of like nodding along because they don't want to interrupt the physician. But maybe they feel more comfortable, you know, stopping and asking a nurse or a nurse practitioner, what do you mean? - I have no idea what you're talking about, kind of thing. And that could be useful, sort of if somebody's having nurse practitioner visits, you know, kind of separate from when the MD is the in room, to again clarify." MDS5  "Well, we have a couple of ways that we educate patients, and one is that when we first make a diagnosis of Parkinson’s... and they're still in the early stages, or somebody else makes the diagnosis and they're still in the early stages...we have a new patient educational clinic [run by our advanced practice nurse practioner]. So we’ll make the diagnosis and then we’ll try to have them come back to this educational session in the next couple of weeks, or at least in the next month. And then at that time – yeah, at that time we’ll go over their individual situation – essentially a group session where we have four, or up to four, new folks, newly-diagnosed folks. And by listening to the other concerns, then they learn a lot about the disease, and even if they're not having that symptom themselves. So that – we spend a lot of time at that session. At that session we do introduce the off-on phenomena, but we don’t spend a – we introduce that and the term dyskinesia and what that means and how it’s different from tremor. So in that way, we educate them about it." MDS7  "I think the way that medicine needs to go, is because of time restraints in general, for patients to get better educated about the disease, it’s hard to depend on the movement disorder neurologist to go over everything in detail. Long time ago... I had the patient see the APP in between every visit that the patient sees me, so that they get that educational piece. So I think that it would be nice if we had a formal way to do continuing education. And presently ...we’ve got the new patient educational thing going – up and going a couple of years ago, and I think now we need to focus – now we’ve got three good APPs though, we can start having a defined education program. We also have another thing we do, and that’s an annual PD clinic. And at that, they see a PT, an ST and an OT, and that includes getting a brief cognitive exam, and also they see the movement disorders neurologist, to wrap it all up." MDS7  "We have an advanced practice provider who, I haven’t used her yet, but many people will alternate doctor and nurse practitioner visits for their more difficult patients because she has an hour and the nursing approach to patients is completely different from the medical approach to patients. I can see the utility for that but there’s no way that we can fund a nurse and a doctor. We just don’t have that capability." MDS8  "Phone calls are handled by a nurse who has a huge knowledge set. A lot of that interaction is predominantly with nurses. I think that people could benefit from training and seeing nurses; it’s just not fiscally possible in a lot of settings." MDS8  "In a perfect world we would all have a social worker, nurse, maybe even a speech pathologists, a physical therapist, a true multidisciplinary clinic and it’s just that in, I’m in a reimbursement office and it’s very difficult to do. There’s no way to fund it." MDS8  "One is education and that would be education through my nurse practitioner or my, you know, sort of ancillary staff." MDS9  "I think that, you know, it really takes a village to help with Parkinson's. So, having good medical assistance or a good receptionist or a good nurse practitioner or PA can really be a nice extension of the clinic itself….The nurse practitioner we use for educational purposes and a lot of our more advanced patients that require deeper [in] stimulation or anything else they'll use - the nurse practitioner will see them sometimes more frequently than they see me." MDS9  "Definitely having the interdisciplinary team improved communication and we do kind of have an interdisciplinary clinic here where they get to, they have the nursing, an actual nursing staff and physical therapy and social work and all those things. So they have opportunities to communicate different problems with different people " MDS10 |
| Patients with different levels of interest and insight may benefit from different types of educational tools | "I think that that would sort of determine how to tailor the handout, or the literature. You know, whether you're dealing with somebody who needs a more nitty gritty in terms of this is what's happening in your brain, or somebody who is like, really basic, like, this is what on looks like. This is what off looks like. Cause I feel like majority of my patients would already know how that feels [motorically]." MDS5 |
